# A Prospective, Multicentered, Randomized, Double-Blind, Placebo-Controlled Clinical Trial of Keluoxin Capsules in the Treatment of Microalbuminuria in Patients with Type 2 Early Diabetic Kidney Disease

**DOI:** 10.1089/jicm.2022.0809

**Published:** 2024-02-14

**Authors:** Jinxi Zhao, Shidong Wang, Xiaoran Li, Guangde Zhang, Yuan Xu, Xianling Zheng, Jian Guo, Zhenxian Zhang

**Affiliations:** ^1^ Department of Nephropathy and Endocrinology, Dongzhimen Hospital of Beijing University of Chinese Medicine, Beijing, China.; ^2^ Department of Endocrinology, Xiyuan Hospital of China Academy of Chinese Medical Sciences, Beijing, China.; ^3^ Department of TCM Diabetes, China-Japan Friendship Hospital, Beijing, China.; ^4^ Department of Endocrinology, Handan Central Hospital, Handan, China.; ^5^ Department of Endocrine and Metabolic Diseases, Tianjin Hospital of ITCWM Nankai Hospital, Tianjin, China.; ^6^ Diabetes Clinic, Luohe Hospital of Traditional Chinese Medicine, Luohe, China.

**Keywords:** diabetic kidney disease, Keluoxin capsules, microalbuminuria, randomized controlled trial

## Abstract

**Background:**

To evaluate the efficacy and safety of Keluoxin (KLX) capsules and provide validated evidence for the application of KLX in the treatment of diabetic kidney disease (DKD).

**Methods:**

A multicenter, randomized, double-blind, placebo-controlled trial design was used to screen 129 patients with DKD (urinary albumin-to-creatinine ratio [UACR]: male, 2.5–30 mg/mmol; female, 3.5–30 mg/mmol) and with Qi and Yin deficiency and blood stasis symptoms. Written informed consent was obtained from all patients. The patients were randomly divided into KLX and control groups. The KLX group was orally administered KLX (6 g/day) and irbesartan tablets (150 mg/day), whereas the control group was administered KLX placebo (6 g/day) and irbesartan tablets (150 mg/day). Patients were observed for 24 weeks to evaluate the natural logarithm of the UACR (log-UACR), the odds ratio (OR) for a sustained increase in the UACR of at least 30% and 40%, estimated glomerular filtration rate (eGFR), changes in symptoms and quality–of-life scores, and adverse events.

**Results:**

The changes of the natural log-UACR during the 24 weeks compared with baseline in the KLX group were better than those in the control group (LS mean ± standard error, −0.26 ± 0.10 vs. 0.01 ± 0.09, *p* = 0.0292). The incidence of a sustained increase in the UACR of at least 30% and 40% was found to be significantly lower in the KLX group (OR, 0.26; 95% confidence interval [CI], 0.09–0.75; OR, 0.29; 95% CI, 0.10–0.82). Changes in symptoms and quality-of-life scores in the KLX group were better than those in the control group. There was no statistically significant difference in eGFR or the incidence of adverse events between the groups.

**Conclusions:**

Overall, these results suggest that KLX capsules combined with irbesartan can reduce microalbuminuria, relieve the symptoms, and improve the quality of life for patients with type 2 early DKD compared with the use of irbesartan alone.

**Trial registration:**

Chinese Clinical Trial Registry, registration number: ChiCTR2100052764.

## Introduction

Diabetic kidney disease (DKD) is a major microvascular complication of diabetes mellitus. According to statistics, ∼20–40% of patients with diabetes mellitus develop chronic kidney disease (CKD).^1^ DKD is the main cause of end-stage renal disease and renal replacement therapy.^2^ DKD greatly reduces the quality of life of patients and significantly increases their medical economic burden.^3–5^

The main clinical manifestations of DKD are an increase in urinary protein and/or a decrease in glomerular filtration rate (GFR). Most patients progress from microalbuminuria and a normal or high GFR to massive proteinuria and a decrease in GFR.^6^ Proteinuria indicates podocyte injury, glomerular basement membrane abnormality, and endothelial cell damage,^7^ and is an important risk factor for the progression of DKD, the occurrence of cardiovascular events, and an increase in all-cause mortality.^8–10^ Several guidelines recommend mainly determining proteinuria excretion using the urinary albumin-to-creatinine ratio (UACR) of random urine and early screening and treatment to delay the progression of DKD.^11^

Angiotensin-converting enzyme inhibitors, angiotensin II receptor blockers (ARB), sodium-glucose cotransporter-2 inhibitors (SGLT-2i), and glucagon-like peptide-1 receptor agonists (GLP-1RA) have attracted extensive attention and have been clinically applied in the treatment of early DKD. They can delay the progression of DKD by reducing urinary proteins to provide a degree of kidney protective benefits.^12,13^ However, they cannot prevent the deterioration of DKD or the occurrence of end-stage renal disease. In clinical treatment, they are sometimes limited by adverse reactions such as hyperkalemia, acute kidney injury, and urinary tract infection; thus, this treatment strategy cannot be fully implemented. Therefore, further efforts are required to explore new drugs to better meet the clinical treatment needs for DKD.

Traditional Chinese Medicine (TCM) has been widely used for the treatment of DKD in China and other Asian regions. Studies have indicated that TCM reduces proteinuria and protects the kidneys.^14^ Owing to the different pharmacological mechanisms of Western medicine, TCM can be complementary to Western medicine in clinical applications.^15,16^ TCM is based on Qi and Yin deficiencies being the basic syndromes of early DKD, and blood stasis is a factor of the disease.^17^

Keluoxin (KLX) capsules, based on the theory of TCM, invigorate Qi with Astragali radix (dried root of *Astragalus mongholicus* Bunge) and Pseudostellariae radix (dried root tuber of *Pseudostellaria heterophylla* (Miq.) Pax), nourish Yin with Lycii fructus (dried mature fruits of *Lycium barbarum* L.) and Ligustri lucidi fructus (dried mature fruits of *Ligustrum lucidum* W.T.Aiton), and promote blood circulation and improve blood stasis with Rhei radix et rhizome (dried root and rhizome of *Rheum officinale* Baill.) and Hirudo (dried body of *Hirudo nipponica* Whitman).

KLX is the first Chinese patent medicine approved for the treatment of DKD (National Drug Standard approval no. Z20090035) and is recommended in several Chinese DKD guidelines.^18,19^ Previous pharmacological studies have shown that KLX reduces podocyte damage by promoting lysosomal degradation to regulate podocyte autophagy.^20^

In addition, KLX synergistically increases antioxidant capacity in combination with enalapril.^21^ Previous clinical research results on DKD patients with microalbuminuria showed that KLX combined with ARB may have a better curative effect than ARB alone in reducing proteinuria and protecting renal function,^22^ but there is still a lack of high-quality clinical evidence to support this. To test this hypothesis, a prospective, multicenter, randomized, double-blind, placebo-controlled clinical trial was conducted to evaluate the efficacy and safety of KLX in the treatment of early DKD with the aim of providing high-quality research evidence of KLX for the treatment of DKD.

## Materials and Methods

### Trial design

This prospective, multicenter, randomized, double-blind, placebo-controlled trial was conducted at six centers: Dongzhimen Hospital of Beijing University of Chinese Medicine, Xiyuan Hospital of China Academy of Chinese Medical Sciences, China-Japan Friendship Hospital, Handan Central Hospital, Tianjin Hospital of ITCWM Nankai Hospital, and Luohe Hospital of Traditional Chinese Medicine. The total cohort size was 120, including 60 patients in the KLX group and 60 in the control group. The trial was retrospectively registered in the Chinese Clinical Trial Registry (www.chictr.org.cn/; registration number: ChiCTR2100052764).

### Exclusion criteria

The exclusion criteria were as follows: (1) Patients who were diagnosed with diabetes mellitus type 1, renal artery stenosis, or renovascular hypertension; (2) Pregnant or lactating women, or those undergoing family planning; (3) Patients with disabilities prescribed by law (blind, deaf, mute, or physical disability); (4) Patients with mental and intellectual disabilities; (5) Patients with severe heart and lung diseases and chronic liver and kidney dysfunction, including those with blood ALT and AST levels higher than the upper normal limits; (6) Patients who are allergic to the ingredients of the experimental drug or those with an allergic constitution; (7) Patients who cannot accept a diet for DKD; (8) Patients who have suffered from malignant hypertension, acute myocardial infarction, cerebrovascular accident, diabetic ketoacidosis, and other critical illnesses, and patients who have congestive heart failure I–IV or chronic diarrhea; (9) Those whose urine microalbumin or proteinuria excretion rate increased due to primary or secondary renal diseases (chronic nephritis, hypertension, gout, and lupus erythematosus); (10) Those who have participated in other clinical trials within 1 month before enrolment.

### Inclusion criteria

This study included patients aged 18–70 years who met the diagnostic criteria for diabetes mellitus type 2 and DKD. Requirements were as follows: (1) Blood pressure, < 140/90 mmHg; (2) Glycosylated haemoglobin, ≤7.5%; (3) UACR, male 2.5–30 mg/mmol, female 3.5–30 mg/mmol; (4) TCM syndrome differentiation exhibits symptoms of Qi and Yin deficiency with blood stasis (In addition to the main symptoms of fatigue and dry mouth and throat, the disease exhibits at least one of the following three symptoms: limb numbness and pain, aggravated at night; lip and tongue are light to dark purple, with ecchymosis; squamous and dry skin. Combined tongue image (the tongue is light to dark purple or has petechiae, ecchymosis, or sublingual collaterals are purple and dilated). Pulse condition (the pulse is thin, both thin and stingy, or both thin and astringent), as it should be diagnosed); (5) Voluntary participation in this clinical study and provision of written informed consent.

### Intervention

The patients received 2 weeks of basic treatment in the lead-in period and 24 weeks of experimental intervention treatment in turn. After the lead-in period treatment, another evaluation was performed, and the subjects who met the inclusion criteria entered the experimental intervention treatment stage.

The basic treatment plan for the lead-in period was formulated according to the 2014 ADA Diabetes Kidney Disease Consensus,^23^ and included: (1) nutrition therapy: a high-quality low-protein diets for patients with diabetes mellitus, and protein intake <0.8 g/kg·day; (2) hypoglycemic therapy: oral medications such as gliquidone, repaglinide and acarbose or combined with insulin are used to control the target HbA1c <7.5%; (3) antihypertensive treatment: irbesartan tablets (150 mg/day, orally), combined accordingly with dihydropyridine calcium antagonists or β receptor blockers, with target blood pressure <130/80 mmHg; (4) lipid-lowering therapy: statins and fibrates were used to control low-density lipoprotein to be <700 mg/L; and (5) diet, exercise, and psychological guidance as required.

The treatment plan in the experimental intervention stage was based on the basic treatment in the lead-in stage: the treatment group was orally administered KLX (4 capsules/time, 3 times/day), orally, the control group was treated with KLX placebo 4 capsules/time, 3 times/day, orally. In addition to experimental drugs, other Chinese medicines, Western medicines, and other treatments related to the treatment of early DKD were prohibited during the lead-in period and experimental intervention stage.

### Experimental drug

The experimental drug was KLX capsules (Chengdu Kanghong Pharmaceutical Co., Ltd.), with specification: 0.5 g/capsule, and code number approved by the SFDA: Z20090035. KLX was composed of Astragali radix, Pseudostellariae radix, Ligustri lucidi fructus, Lycii fructus, Rhei radix et rhizome, and Hirudo. Two studies have analyzed the chemical constituents and blood transitional ingredients of KLX capsules by UPLC-Q-TOF-MS technology.^24,25^ In addition, 212 chemical compounds were identified from KLX, including anthraquinones, flavonoids, terpenoids, and taxonomic compounds.^25^ The control drug was KLX capsule simulant (Chengdu Kanghong Pharmaceutical Co., Ltd.), with specification: 0.5 g/capsule. The shape, packaging, taste, color, and other characteristics of the experimental drug and placebo were consistent.

Basic medication was irbesartan tablets (Sanofi [Hangzhou] Pharmaceutical Co., Ltd.), with specification: 0.15 g/tablet and code number approved by the SFDA: H20040494. The experimental drug administrator at each center was responsible for the storage, distribution, recovery, and recording of drugs. A special counter was set up to store the experimental drug, which was kept ventilated and dry at an appropriate temperature. After the drug was used for 24 weeks, all remaining drugs were recovered and returned to the sponsor.

### Outcome measures

The primary measure of effectiveness was the natural logarithm of the UACR (log-UACR) and the odds ratio (OR) for the sustained increase in the UACR of at least 30% and 40%, and the secondary measures included the following: (1) TCM symptom score (Supplementary File S1 for the score table); (2) quality-of-life assessment, including the world health organization quality of life-bref (WHOQOL-BREF) and the scale of diabetes quality of life (DQOL) (Supplementary File S2); (3) estimated glomerular filtration rate (eGFR), estimated using the modified MDRD formula for Chinese patients with CKD (eGFR = 175 × SCr^−1.234^ × Age^−0.179^ × [0.79, female] or [1, male]); and (4) clinical and laboratory monitoring measures: blood pressure, blood glucose (fasting, postprandial blood glucose, and HbA1c), and blood lipids (cholesterol, triglycerides, high-density lipoprotein, and low-density lipoprotein).

Safety observation measures included the following: (1) general physical examination items: heart rate, respiration, blood pressure, body temperature, and body mass index (BMI); (2) blood routine, urine routine, and electrolytes (potassium, sodium, chlorine, calcium, and phosphorus); (3) liver function (ALT, AST, TBIL, ALP, and r-GT), renal function (BUN and Cr); (4) ECG examination, B-ultrasound (liver, gallbladder, spleen, pancreas, and both kidneys), and fundus examination; (5) detailed information about any adverse event, including the rate of incidence and correlation with the treatment, were recorded.

### Visits and data collection

A total of five visits were arranged, including enrolment visits (week −2), baseline visits (week 0), and intervention visits (week 4 ± 2 days, week 12 ± 4 days, and week 24 ± 6 days). In addition to general physical examination information (heart rate, respiration, blood pressure, body temperature, and BMI) and combined medication, the TCM symptom score, quality–of-life assessment, and adverse events were recorded at baseline and during all treatment visits. The UACR and eGFR were measured during all visits. Fundus examinations were performed during the baseline visit.

Liver and kidney functions, including transaminase, bilirubin, GGT, cholinesterase, urea nitrogen, and creatinine levels, were monitored at baseline and during all treatment visits. Therefore, blood routine, urine routine, blood lipid, electrolyte (potassium, sodium, chlorine, calcium, and phosphorus), fasting and 2 h postprandial blood glucose, HbA1c, abdominal B-ultrasound, and ECG were completed at baseline and during all treatment visits during the 24 weeks. After each visit, the writing of research medical records and the entry for electronic data collection were completed.

### Sample size calculation

This was an exploratory study, using an optimal experimental design based on the relevant literature and previous research results of KLX and irbesartan.^26,27^ The UACR, compared with the baseline of the control group, decreased by 40 mg/g after irbesartan tablets + basic treatment for 24 weeks. Assuming that the UACR compared to the baseline of KLX + irbesartan + basic treatment for 24 weeks decreased by 65 mg/g, the standard deviation (SD) was 50, the difference between groups of UACR for 24 weeks was 25, the class I error was 10% on both sides, and the statistical efficiency was 80%. In total, 51 patients were required in each group; thus, 60 patients were required considering ∼15% shedding. Therefore, a total of 120 patients were required.

### Randomization and allocation concealment

The CRO company, independent of the trial, generated a random drug code according to the hierarchical and segmented method using SAS software. After the drug code was divided into an experimental group and a control group, it was successively assigned to six branch centers, and the grouping information was recorded in the random allocation result, which was hidden by the team leader unit and sponsor. The statistician repacked and labeled the drugs according to the random allocation results and created an emergency envelope for emergency unblinding.

During the trial, the patients and researchers were blinded to the grouping situation. After blinding verification, the data were locked and the first unblinding was performed. The groups were informed of the statisticians, with A and B as codes. When the statistical analysis was completed and the statistical report was completed, a second unblinding was performed, and the exact groups A and B were announced. Finally, a summary of the clinical trial was presented. In the process of a clinical trial, if all the random allocation results were leaked or the opening rate of emergency letters exceeded 20%, the double-blind trial would be invalid.

### Quality control and ethical principles

This trial was monitored by Beijing Kangpaixing Pharmaceutical Technology Development Co., Ltd., which ensured that the researchers had the professional expertise, qualification, and ability of clinical trials, and ensured that each center made the researchers fully understand the clinical trial protocol and the specific connotation of each measure through preclinical trial training. The researchers tested the consistency of the quantitative standards of symptoms and signs at different centers. Induction or prompts were prohibited. The specified objective measures were checked according to the time point and method specified in the protocol, and the laboratories of all participating parties were tested according to standard operating and quality control procedures.

The study protocol was approved by the Medical Ethics Committee of Dongzhimen Hospital, Beijing University of Chinese Medicine, before the start of the trial (approval no.: ECPJ-BDY-2014-24). During the trial, the patients were fully informed of the trial, their privacy was protected, and the rights requirements of the patients stipulated in the *World Medical Association Declaration of Helsinki* were met.

### Statistical analysis

The full analysis set (FAS) and per-protocol set (PPS) were used to analyze the efficacy measures, and the mixed models for repeated measures were used to compare the changes and change rate of log-UACR and total score of TCM symptoms between the two groups at weeks 4, 12, and 24, and to compare the changes in blood glucose, blood lipid, blood pressure, and quality-of-life scale scores between the two groups during the 24 weeks. A logistic regression model was used to analyze the OR for a sustained increase in UACR. The comparison of the two categorical variables between the two groups was performed using the *χ*^2^ test or Fisher test; the comparison of ordered multiclass variables between the two groups was performed using the Wilcoxon rank-sum test.

For the comparison of other secondary efficacy measures such as quality-of-life score, serum creatinine, and blood lipid indicators between the two groups, based on whether they obeyed normal distribution, *t*-test, covariance test, or Wilcoxon rank-sum test, the *t*-test, covariance test, or Wilcoxon rank-sum test was used. Comparisons within groups were performed using the *t*-test or Wilcoxon rank-sum test.

The comparison of eGFR between the two groups and within groups was performed using the *t*-test or Wilcoxon rank-sum test. The safety set (SS) was used to analyze all safety data, and treatment-related adverse event data were listed according to the number of patients with at least one event, the percentage of patients with at least one event, the number of adverse events, and the incidence of adverse events. The *χ*^2^ test/Fisher test was used to compare the incidence of adverse events between the two groups, and the adverse events in this trial are listed.

## Results

### Patient process and baseline characteristics

**FIG. 1.**
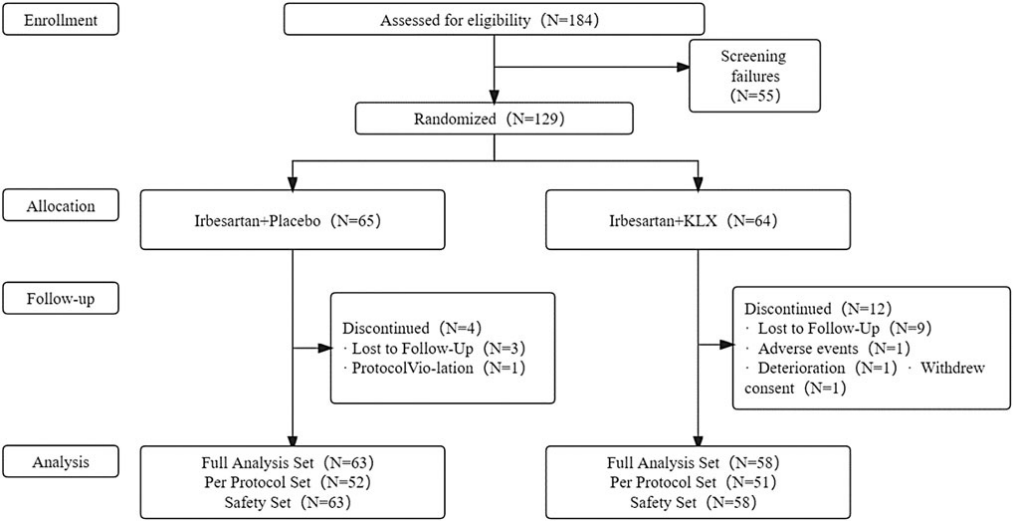
Flow diagram of patient inclusions.

Between June 2014 and September 2019, 129 patients completed the screening. Among them, 65 and 64 were randomly assigned to the control and experimental groups, respectively. There were 16 cases of shedding in the study, and 113 patients completed the treatment, including 61 cases in the control group and 52 cases in the experimental group. In the statistical analysis, the FAS included 121 cases, PPS included 103 cases, and SS included 121 cases. Further details are provided in Figure 1. There was no statistically significant difference in age, sex, duration of diabetes, BMI, heart rate, UACR, eGFR, or HbA1c between the two groups at baseline (*p* > 0.05), as shown in Table 1.

**Table 1.** Baseline Characteristics of Patients

	KLX + irbesartan (*n* = 58)	Placebo + irbesartan (*n* = 63)	*p*
Age (years)	56.75 (9.50)	57.55 (9.14)	0.6386
Gender (M/F)	39/19	36/27	0.2530
Duration of diabetes (month)	29.96 (42.19)	23.36 (26.62)	0.9404
BMI (kg/m^2^)	26.02 (3.54)	26.24 (3.37)	0.7269
HR (bpm)	74.09 (6.60)	74.79 (6.13)	0.5423
UACR (mg/g)	96.83 (72.45)	91.56 (67.64)	0.8295
eGFR (mL/min/1.73 m^2^)	110.99 (32.39)	111.53 (31.09)	0.9271
HbA1c (%)	6.6 (0.69)	6.7 (0.65)	0.1629

Note: sex, expressed in quantity; and others, expressed as mean (SD).

BMI, body mass index; eGFR, estimated glomerular filtration rate; HbA1c, glycosylated hemoglobin; HR, heart rate; KLX, Keluoxin; SD, standard deviation; UACR, urinary albumin-to-creatinine ratio.

### Primary outcome measures

The log-UACR value in the KLX group gradually decreased during weeks 4, 12, and 24. The log-UACR values in the control group at all visit points were similar to baseline values. The change of log-UACR in the KLX group was −0.26 ± 0.10 by week 24, and the change rate was −6.29 ± 2.33%. The change in the control group was 0.01 ± 0.09, and the change rate was 0.93 ± 2.17%. There was a significant difference in the changes and change rates between the groups (*p* < 0.05). Further details are provided in Table 2. A sustained increase in the UACR of at least 30% occurred in six patients in the KLX group and 19 patients in the control group (OR, 0.26; 95% confidence interval [CI], 0.09–0.75). The outcome of a sustained increase in the UACR of at least 40% occurred in six patients in the KLX group and 18 patients in the control group (OR, 0.29; 95% CI, 0.10–0.82). Further details are provided in Table 3.

**Table 2.** Comparison of the Changes of Log-Urinary Albumin-to-Creatinine Ratio Between the Keluoxin and Control Groups

	KLX + irbesartan	Placebo + irbesartan	*p*
Changes of log-UACR compared with the baseline, LS mean (SE)			
Week 4	−0.12 (0.09)	−0.02 (0.09)	0.3913
Week 12	−0.21 (0.09)	−0.05 (0.09)	0.1767
Week 24	−0.26 (0.10)	0.01 (0.09)	**0.0292**
Change rate of log-UACR compared with the baseline (%), LS mean (SE)			
Week 4	−2.64 (2.21)	−0.12 (2.10)	0.3706
Week 12	−4.71 (2.25)	−1.15 (2.13)	0.2151
Week 24	−6.29 (2.33)	0.93 (2.17)	**0.0154**

Bold indicates *p* < 0.05.

Note: the changes in log-UACR compared with the baseline and the change percentage of log-UACR compared with the baseline were analyzed using the MMRM. The KLX group (baseline *n* = 58, week 4 *n* = 55, week 12 *n* = 53, week 24 *n* = 48) and the control group (baseline *n* = 63, week 4 *n* = 62, week 12 *n* = 59, week 24 *n* = 56).

log-UACR, natural logarithm of the UACR; LS, least squares; MMRM, mixed models for repeated measures; SE, standard error.

**Table 3.** Comparison of Outcomes of a Sustained Increase in the Urinary Albumin-to-Creatinine Ratio

	KLX + irbesartan	Placebo + irbesartan	OR (95% CI)
	No./total no.	No./total no.	
Increase in UACR of at least 30%	6/48	19/56	0.26 (0.09–0.75)
Increase in UACR of at least 40%	6/48	18/56	0.29 (0.10–0.82)

CI, confidence interval; OR, odds ratio.

### Clinical and laboratory examination

There was no statistically significant difference between the groups in the changes in systolic blood pressure, diastolic blood pressure, total cholesterol, HbA1c, eGFR, and low-density lipoprotein by week 24 compared with baseline. Compared with the baseline changes of triglyceride, the KLX group had better outcomes than the placebo group, and the difference between the groups was −0.4 ± 0.22 mmol/L, which was statistically significant (*p* < 0.05). Further details are provided in Table 4.

**Table 4.** Comparison of Changes Between the Keluoxin and Control Groups

	KLX + irbesartan		Placebo + irbesartan		Inter-group differences of change from baseline to week 24	*p*
	Baseline* n* = 58	Week 24* n* = 48	Baseline* n* = 63	Week 24* n* = 56		
	Mean (SD)	Mean (SD)	Mean (SD)	Mean (SD)	LS mean (SE)	
Systolic pressure (mmHg)	127.5 (7.98)	125.8 (6.84)	128.4 (9.03)	127.9 (8.02)	−1.6 (1.09)	0.136
Diastolic pressure (mmHg)	78.4 (7.05)	76.6 (6.73)	77.2 (7.13)	77.8 (6.43)	−1.1 (0.97)	0.276
eGFR (mL/min/1.73 m^2^)	110.99 (32.39)	110.24 (33.98)	111.53 (31.09)	104.96 (28.16)	3.55 (3.48)	0.3084
HbA1c (%)	6.6 (0.69)	7.3 (1.57)	6.7 (0.65)	7.1 (1.41)	0.2 (0.28)	0.383
TC (mmol/L)	4.6 (1.06)	4.5 (0.98)	4.5 (0.93)	4.7 (0.90)	−0.1 (0.16)	0.389
TG (mmol/L)	2.0 (1.40)	1.7 (0.82)	1.8 (1.05)	2.1 (1.90)	−0.4 (0.22)	**0.043**
LDL-C (mmol/L)	2.6 (0.84)	2.7 (0.83)	2.7 (0.76)	2.8 (0.74)	−0.1 (0.13)	0.650

Bold indicates *p* < 0.05.

Note: analysis of covariance was used for intergroup changes and intergroup *p* value.

LDL-C, low-density lipoprotein; TC, total cholesterol; TG, triglyceride.

### Symptom score

There was no between-group difference in the disappearance rates of the main symptoms and symptoms of fatigue and dry mouth and throat at weeks 4 and 12. For the KLX group, the disappearance rate of the main symptoms by week 24 was 23.08% (95 CI 12.53%–36.84%), which was higher than the 8.20% (95 CI 2.72%–18.10%) in the control group. The disappearance rates of single symptoms of fatigue and dry mouth in the KLX group by week 24 were 36.54% (95 CI 23.62%–51.04%) and 50.00% (95 CI 35.81%–64.19%), respectively, which were higher than 18.03% (95 CI 9.36%–29.98%) and 27.87% (17.15% to 40.83%) in the control group (*p* < 0.05).

The total symptom score of the KLX and control groups decreased gradually by weeks 4, 12, and 24. The changes in the total symptom score of the KLX group by week 12 were as follows: LS mean ± standard error: −5.18 ± 0.44, which was better compared with the control group, −3.15 ± 0.42. The change of the total symptom score of the experimental group at week 24 was −7.24 ± 0.45 points, which was better compared with the control group, −4.61 ± 0.42. The difference between the groups was statistically significant (*p* < 0.05). Further details are provided in Table 5.

**Table 5.** Comparison of Changes of Main Symptoms and Symptom Scores

	KLX + irbesartan	Placebo + irbesartan	*p*
Disappearance rate of main symptoms (%), mean (95 CI)			
Week 4	7.02 (1.95–17.00)	1.59 (0.04–8.53)	0.3034
Week 12	5.45 (1.14–15.12)	3.23 (0.39–11.17)	0.8911
Week 24	23.08 (12.53–36.84)	8.20 (2.72–18.10)	**0.0274**
Disappearance rate of fatigue (%), mean (95 CI)			
Week 4	14.04 (6.26–25.79)	7.94 (2.63–17.56)	0.2831
Week 12	16.36 (7.77–28.80)	12.90 (5.74–23.85)	0.5960
Week 24	36.54 (23.62–51.04)	18.03 (9.36–29.98)	**0.0264**
Disappearance rate of dry mouth and throat (%), mean (95 CI)			
Week 4	15.79 (7.48–27.87)	6.35 (1.76–15.47)	0.0966
Week 12	23.64 (13.23–37.02)	14.52 (6.86–25.78)	0.2076
Week 24	50.00 (35.81–64.19)	27.87 (17.15–40.83)	**0.0157**
Total symptom score, mean (SD)			
Baseline	13.03 ± 4.10	12.65 ± 5.05	0.4152
Week 4	10.35 ± 3.87	10.62 ± 4.14	0.9115
Week 12	7.87 ± 2.67	9.76 ± 3.96	**0.0206**
Week 24	5.60 ± 2.72	8.28 ± 4.08	**0.0002**
Changes of total symptom score compared with baseline, LS Mean (SE)			
Week 4	−2.65 (0.43)	−2.25 (0.41)	0.4698
Week 12	−5.18 (0.44)	−3.15 (0.42)	**0.0004**
Week 24	−7.24 (0.45)	−4.61 (0.42)	**0.0001**

Bold indicates *p* < 0.05.

Note: The KLX group (baseline *n* = 58, week 4 *n* = 57, week 12 *n* = 55, week 24 *n* = 52) and the control group (baseline *n* = 63, week 4 *n* = 63, week 12 *n* = 62, week 24 *n* = 61); the changes in the total scores of TCM syndrome were analyzed using the MMRM; disappearance rate of fatigue: the proportion of patients with disappearance of fatigue; disappearance rate of dry mouth and throat: the proportion of patients whose symptoms of dry mouth and throat disappeared; disappearance rate of main symptoms: the proportion of patients whose symptoms of fatigue, dry mouth, and throat disappeared.

TCM, Traditional Chinese Medicine.

### Quality of life

There was no statistical significance in the total scores of WHOQOL-BREF and DQOL scales between the experimental and control groups at baseline, but the total scores of WHOQOL-BREF in the KLX group increased to mean ± SD: 59.92 ± 6.01 at week 24, which was higher compared with the control group at mean ± SD: 57.5 ± 6.24 in (*p* < 0.05).

The total score of DQOL in the KLX group decreased to mean ± SD: 47.84 ± 9.62 at week 24, which was significantly lower compared with the control group at mean ± SD: 51.65 ± 10.06 (*p* < 0.05). In the WHOQOL-BREF scale, the improvement in the scores in the fields of physiology, social relations, and environment of the KLX group was greater compared with the control group. In the DQOL scale, the improvement in the scores in the field of physiological function and psychology/spirit of the KLX group was greater compared with the control group. Further details are provided in Table 6.

**Table 6.** Changes of Quality-of-Life Scores in the Keluoxin and Control Groups

	KLX + irbesartan	Placebo + irbesartan	*p*
WHOQOL-BREF scale			
BREF total scores (baseline), mean (SD)	57.85 (6.22)	57.37 (6.30)	0.6776
BREF total scores (week 24), mean (SD)	59.92 (6.01)	57.50 (6.24)	**0.0450**
Change rate of physiological field score by week 24 (%), LS Mean (SE)	4.27 (1.36)	−0.22 (1.27)	**0.0094**
Change rate of psychological field score by week 24 (%), LS mean (SE)	2.96 (1.34)	0.26 (1.26)	0.1108
Change rate of social relations field score by week 24 (%), LS mean (SE)	6.16 (1.31)	2.87 (1.23)	**0.0485**
Change rate of environmental field score by week 24 (%), LS mean (SE)	4.45 (1.32)	−0.16 (1.24)	**0.0060**
DQOL scale			
DQOL scores (baseline), mean (SD)	52.24 (10.65)	54.33 (12.03)	0.3084
DQOL scores (week 24), mean (SD)	47.84 (9.62)	51.65 (10.06)	**0.0082**
Change rate of physiological function score by week 24 (%), LS mean (SE)	−7.79 (2.25)	−1.91 (2.12)	**0.0407**
Change rate of psychological/mental score by week 24 (%), LS mean (SE)	−7.39 (1.69)	−2.92 (1.58)	**0.0386**
Change rate of social relations score by week 24 (%), LS mean (SE)	−2.76 (2.38)	3.15 (2.19)	0.0518
Change rate of patient impact score compared with the baseline by week 24 (%), LS mean (SE)	−3.35 (2.85)	0.38 (2.66)	0.3050

Bold indicates *p* < 0.05.

Note: The KLX group (baseline *n* = 58, week 24 *n* = 52) and the control group (baseline *n* = 63, week 24 *n* = 61); the score change rate of the scale was analyzed using the MMRM.

DQOL, diabetes quality of life; WHOQOL-BREF, the world health organization quality of life-bref.

### Adverse events

There was no statistically significant difference in the number and incidence of adverse events between the KLX and control groups during the study period (*p* > 0.05). Adverse events mainly involved respiratory tract infection, urinary tract infection, abnormal liver function, dizziness, nausea, and limb pain. Further details are provided in Supplementary File S3. There was no statistically significant difference between the two groups in the number of cases of withdrawal due to adverse events and drug reactions (*p* > 0.05). No serious adverse event occurred in either group. Further details are provided in Table 7.

**Table 7.** Analysis of Adverse Events/Reactions of Patients During the Trial

	KLX + irbesartan (*n* = 58)			Placebo + irbesartan (*n* = 63)			Statistic	*p*
	Times	Number	Occurrence rate	Times	Number	Occurrence rate		
Adverse events	26	20	34.48%	27	19	30.16%	*χ*^2^ = 0.2585	0.6112
Serious adverse events	0	0	0.00%	0	0	0.00%	—	—
Withdrawal due to adverse events	3	2	3.45%	0	0	0.00%	Fisher	0.2277
Adverse reaction	5	3	5.17%	7	6	9.52%	Fisher	0.4944

Note: Number, one patient counts at most once; Times, one patient counts according to the actual occurrence times; *n*, total number of patients in each group in the safety analysis set; %, the percentage of adverse events in the total number of patients in each group; relationship with drugs, must be related, may be related, and unable to be determined can be regarded as adverse reactions to the drug.

## Discussion

Several recent large-scale studies on drugs for CKD related to type 2 diabetes showed significant improvement in albuminuria mainly in the baseline macroalbuminuria group, while the effect on the microalbuminuria or normoalbuminuria groups was unknown.^28–30^ Previous studies have shown that KLX capsules can inhibit inflammatory reaction, protect renal function, and delay the progression of DKD in patients with microalbuminuria.^31–33^

As part of the first batch of Chinese patent medicines approved for the treatment of DKD, KLX capsules have accumulated some clinical evidence of its combination with ARB in the treatment of DKD in the early stage. However, research quality and evidence intensity are generally low. This was a double-blind, multicenter randomized clinical trial, and on the basis of the standard treatment of low-dose irbesartan (150 mg/day), KLX capsules or placebo was administered to verify the efficacy and safety of KLX capsules in the treatment of early DKD with Qi and Yin deficiency and blood stasis. Our study has preliminarily indicated the potential refined treatment plans for patients with microalbuminuria.

The results showed that the log-UACR of the KLX capsule group was additionally reduced during week 24, and patients in the KLX capsules group had a lower risk of sustained increase in the UACR. A previous clinical study found that the combination of KLX and irbesartan in treating DKD can reduce proteinuria quantification for another 24 h compared to irbesartan alone.^34^ Collectively, with the aforementioned study, this study has verified the therapeutic effect of KLX capsules on reducing proteinuria in early DKD.

In contrast, the UACR used in this study is more suitable for evaluating changes in proteinuria in early DKD.^35^ Previous pharmacological mechanism studies have indicated that the clinical benefit of KLX capsules in reducing proteinuria was related to the pharmacological effect of regulating podocyte autophagy and reducing podocyte injury.^20^ In addition, as the main ingredients of KLX, Astragalus was reported to inhibit the overexpression of eNOS; leeches can inhibit the expression of NOX4 in the kidney, and Fructus Lycii can regulate Nuclear factor kappa B (NF-κB). All these factors can help to enhance antioxidant capacity and reduce proteinuria.^36–38^ This pharmacological action may be the possible reason for the reduction in proteinuria in DKD by KLX capsules.

It is noteworthy that RAS blockers can reduce proteinuria and also regulate Nrf2, inhibit transforming growth factor-β, and exert the renal protective function of antioxidant stress.^39^ The intersection of pharmacological mechanisms between the two and the mechanism of combined medications require further study in the future. In addition, the blood pressure, blood glucose, and blood lipid levels in this study after 24 weeks of treatment were consistent with the baseline values. The study found that the experimental group may have advantages in reducing triglycerides, which is expected to be further explored in the design and control of lipid-lowering drugs in the future.

In this trial, there was no statistically significant difference in eGFR during the 24 weeks between KLX capsules combined with irbesartan and irbesartan alone. This result may be related to the slow progression of DKD, shorter research period, and high baseline eGFR. It is noteworthy that in this study, during the 24 weeks, the eGFR of the irbesartan group decreased, which is consistent with the conclusion of a previous clinical study of irbesartan; that is, the decrease in proteinuria after irbesartan treatment is related to the eGFR.^40^

However, the combination of KLX capsules reduced the decrease in eGFR, but there was no statistical significance in the degree of slowdown in this study. Fructus Lycii, an ingredient of KLX, can improve renal function and delay the progression of DKD by inhibiting the expression of NF-κB.^41^ Another ingredient of KLX, rhubarb, can inhibit renal fibrosis, promote toxin excretion, improve early symptoms of chronic renal insufficiency, and delay renal failure.^42^ These studies suggest that KLX capsules may have an effect on improving renal function, and more evidence-based medical findings are required through prolonging the research cycle, increasing the sample size, and/or increasing the therapeutic dose of irbesartan.

This study also found that compared with the control group, KLX capsules had better performance in improving the clinical symptoms of patients, such as dry mouth and fatigue. This advantage mainly comes from the treatment concept of TCM syndrome differentiation and treatment. In addition, this study found that KLX capsules were superior to placebo in improving the quality of life of patients with DKD. To the best of our knowledge, this is the first clinical study to evaluate the efficacy of KLX capsules using quality of life as an outcome.

Both scales indicated that KLX could improve the quality of life in the physiological field in patients with DKD. The improvement in the quality of life of patients with DKD should be attributed to patient-centered treatment with TCM.^4^ Therefore, it was speculated that the improvement in quality of life with KLX capsules may be attributed to the therapeutic advantage of KLX in improving clinical symptoms.

Although the new treatments of recent years have provided extra options for patients with DKD, the clinical use is hindered by adverse reactions, including urinary tract infection and increasing risk of cardiovascular events.^43^ For example, SGLT-2i shows cardiovascular and kidney benefits, and plays a renal protective role by improving renal hyperfiltration status, reducing renal hypoxia, and reducing oxidative stress. However, SGLT-2i increases the risks of infection in urinary tract and in reproductive tract at the same time.^44,45^ Bardoxolone methyl can protect renal function by regulating Nrf2 signaling pathway.

However, clinical studies have found that it may increase the risk of cardiovascular events.^46,47^ In addition to reducing urinary protein and delaying the progression of proteinuria, KLX capsules are proved to significantly relieve the clinical symptoms and improve the quality of life for patients, compared with the medicines mentioned above. However, further study is required on the detailed clinical benefit for extended population. From the perspective of safety, KLX may outperform the medicines mentioned above. There was no difference in the incidence of adverse events between the two groups. No serious adverse event occurred in either of the groups. This was consistent with the results of a previous clinical study,^26^ that is, on the basis of basic ARB treatment, the addition of KLX capsules did not increase the incidence of adverse events, which verified the safety of clinical medication with KLX capsules.

The limitations of this study are noteworthy. First, due to the limitation of the study cycle, the DKD composite endpoint event risk, that is, the risk of doubling blood creatinine, end-stage renal disease, or renal death, was not introduced as an evaluation measure of effectiveness and safety. Second, the basic treatment in this study only used ARB to reduce blood pressure. However, SGLT-2i and GLP-1RA, with the same evidence of renal protection, were not included in the basic treatment. Whether the results of this study are applicable to the combination of SGLT-2i or GLP-1RA requires further study. Third, this study only used low-dose irbesartan as the basic treatment. However, low-dose ARB drugs do not play the best role in the treatment of DKD.^40,48^ Therefore, the combined application of different doses of irbesartan can better reveal the curative effect of KLX.

## Conclusions

Under the conditions of this study, compared with irbesartan alone, KLX capsules combined with irbesartan is an effective therapy for type 2 early DKD in reducing microalbuminuria, relieving the symptoms, and improving the quality of life with high safety and little adverse reaction.

## Supplementary Material

Supplemental data

Supplemental data

Supplemental data

## Data Availability

The datasets generated and analyzed during this study are not publicly available because further research on KLX is underway, but are available from the corresponding author upon reasonable request.
