# Supplementary material for: A Prospective, Multicentered, Randomized, Double-Blind, Placebo-Controlled Clinical Trial of Keluoxin Capsules in the Treatment of Microalbuminuria in Patients with Type 2 Early Diabetic Kidney Disease
Source: J Integr Complement Med. 2024 Feb 14;30(2):185–95. doi: 10.1089/jicm.2022.0809 (PMC10884549; doi:10.1089/jicm.2022.0809)
Supplement: Supplemental data [file Supp_FileS1.docx]

**Supplementary 1: TCM Symptom Score Table**

| Main symptoms | Points | No (0) | Mild (2) | Moderate (4) | Severe (6) |
| --- | --- | --- | --- | --- | --- |
| Fatigue |  | No | Decreased energy and endurance, not affect daily life | Mental fatigue, fatigue in general activity | Mental fatigue, fatigue in rest |
| Dry mouth and throat |  | No | Dry throat | Dry throat, thirsty | Dry throat, greatly thirsty |
| Secondary symptoms | Points | No (0) | Mild (1) | Moderate (2) | Severe (3) |
| Limb numbness and pain, aggravated at night |  | No | Limb numbness, occasional tingling | Persistent numbness and pain in hands and feet | Continuous numbness and pain below knee and elbow, difficult to sleep |
| Purple and dark lips and tongue, with ecchymosis |  | No | Slightly dark lips and tongue, little ecchymosis and slightly purple sublingual collaterals | Dark lips and tongue, little ecchymosis and purple sublingual collaterals | Obviously dark lips and tongue, little ecchymosis and obviously purple and dilated sublingual collaterals |
| Squamous and dry skin |  | No | The skin is rough, dry and moist at some parts. | Different parts of the skin are rough, dry, keratinized and desquamated. | The skin is extensively rough, dry, keratinized and desquamated. |
| Dry eyes and dazzling |  | No | Occasionally dry eyes or small dark shadows in front the eyes, no blurring | Frequently dry eyes or many small dark shadows in front the eyes, slight blurring | Unbearable dry eyes, serious blurring and deformation, and large dark shadows in front of the eyes |
| Lethargy |  | No | Short of breath after activities, lethargic | Intermittent short of breath even at rest, lethargic | Continuous short of breath even when quiet, low voice, weak speech |
| Spontaneous sweating |  | No | Sweat slightly when quiet, but increase when moving | Sweat obviously when quiet, sweat more when moving | Sweat a lot when quiet, sweat greatly when moving |
| Feverish sensation over the palm, sole and heart, or sphoria with feverish sensation in chest，palms and soles |  | No | Occasional feverish sensation over the palm, sole and heart or being slightly upset | Frequent feverish sensation over the palm, sole and heart or being obviously upset | Continuous feverish sensation over the palm, sole and heart or fidget |
| Dry stool |  | No | Dry stool, once a day | Dry stool, once two days | Dry stool, once for several days |
| Tongue imagine | | e.g.: dark lips and tongue, ecchymosis and purple and dilated sublingual collaterals | | | |
| Pulse condition | | e.g.: thin or/and stringy or/and pulse | | | |
