# Supplementary material for: A Prospective, Multicentered, Randomized, Double-Blind, Placebo-Controlled Clinical Trial of Keluoxin Capsules in the Treatment of Microalbuminuria in Patients with Type 2 Early Diabetic Kidney Disease
Source: J Integr Complement Med. 2024 Feb 14;30(2):185–95. doi: 10.1089/jicm.2022.0809 (PMC10884549; doi:10.1089/jicm.2022.0809)
Supplement: Supplemental data [file Supp_FileS2.docx]

**Supplementary 2: Quality of Life Scale**

1. WHO Quality of Life, WHOQOL-BREF

(formulated with reference to WHOQOL-BREF)

Table B1

| Personal Information |
| --- |
| 1. Gender □male □female 2. Age □□□years old 3. Birth Date □□□□year□□month□□day 4. Top Education Qualification □primary school □junior high school □senior high school or secondary specialized school □college for professional training □undergraduate college □postgraduate 5. Marital Status □unmarried □married □cohabitation □separation □divorce □bereft of one’s spouse 6. Are you ill now? □Yes □No 7. What are your health problems at present? 8. Occupation □worker □peasant □administrative worker □workers in service industry □intellectual □other |

Filling Instructions

The following questions are to know how you feel about your quality of life, health status and daily activities. Please be sure to answer all the questions. If you are not sure how to answer a question, choose the answer closest to your true feelings.

Please answer all questions according to your own standards, wishes or feelings. Please note that all problems are only your situation in the last two weeks.

For example: do you get the support you need?

| Not at all  1 | Slightly  2 | Moderately  3 | Mostly  4 | Absolutely 5 |
| --- | --- | --- | --- | --- |

**WHOQOL-BREF** Table B2

| Please put a 〓 at the most appropriate number according to the degree of support you have received from others in the past two weeks. If you can get the support you need most of the time, put a 〓 at the number “4”; if you can’t get the support you need at all, put a 〓 at the number “1”. Please read each question and choose the best answer for your situation according to your feeling. | | | | |
| --- | --- | --- | --- | --- |
| 1．(G1) How do you evaluate your quality of life? | | | | |
| Terrible  1 | Bad  2 | Not bad  3 | Good  4 | Very good  5 |
| 2．(G4) Are you satisfied with your health? | | | | |
| Very dissatisfied  1 | Dissatisfied  2 | Neither satisfied nor dissatisfied  3 | Satisfied  4 | Very Satisfied  5 |
| The following questions are about how you feel about something you have experienced in the last two weeks. Please read each question and choose the best answer for your situation according to your feeling. | | | | |
| 3. (F1.4) Do you think pain prevents you from doing what you need to do? | | | | |
| Not at all  1 | Seldom  2 | Sometimes  3 | Always  4 | Quite frequently  5 |
| 4. Do you need medical help in your daily life? | | | | |
| Not at all  1 | Seldom  2 | Sometimes  3 | Always  4 | Quite frequently  5 |
| 5. (F4.1) Do you think life is fun? | | | | |
| Not at all  1 | Seldom  2 | Sometimes  3 | Always  4 | Quite frequently  5 |
| 6. (F24.2) Do you think your life is meaningful? | | | | |
| Not at all  1 | Seldom  2 | Sometimes  3 | Always  4 | Quite frequently  5 |
| 7. (F5.3) Can you concentrate? | | | | |
| Not at all  1 | Seldom  2 | Sometimes  3 | Always  4 | Quite frequently  5 |
| 8. (F16.1) Do you feel safe in your daily life? | | | | |
| Not at all  1 | Seldom  2 | Sometimes  3 | Always  4 | Quite frequently  5 |
| 9. (F22.1) Is your living environment good for health? | | | | |
| Not at all  1 | Seldom  2 | Sometimes  3 | Always  4 | Quite frequently  5 |
| The following questions are about your ability to do something in the last two weeks. Please read each question and choose the best answer for your situation according to your feeling. | | | | |
| 10. (F2.1) Do you have enough energy to deal with your daily life? | | | | |
| Not at all  1 | Seldom  2 | Sometimes  3 | Always  4 | Quite frequently  5 |
| 11. (F7.1) Do you think your appearance is acceptable? | | | | |
| Not at all  1 | Seldom  2 | Sometimes  3 | Always  4 | Quite frequently  5 |
| 12. (F18.1) Do you have enough money? | | | | |
| Not at all  1 | Seldom  2 | Sometimes  3 | Always  4 | Quite frequently  5 |
| 13. (F20.1) Is there all the information you need in your daily life? | | | | |
| Not at all  1 | Seldom  2 | Sometimes  3 | Always  4 | Quite frequently  5 |
| 14. (F21.1) Do you have opportunities for leisure activities? | | | | |
| Not at all  1 | Seldom  2 | Sometimes  3 | Always  4 | Quite frequently  5 |
| 15. (F9.1) How about your ability to act? | | | | |
| Very bad  1 | Bad  2 | Not good  3 | Good  4 | Very good  5 |
| The following questions are about your satisfaction with all aspects of your daily life in the past two weeks. Please read each question and choose the best answer for your situation according to your feeling. | | | | |
| 16. (F3.3) Are you satisfied with your sleep? | | | | |
| Very dissatisfied  1 | Dissatisfied  2 | Neither satisfied nor dissatisfied  3 | Satisfied  4 | Very  Satisfied  5 |
| 17. (F10.3) Are you satisfied with your ability to do things in daily life? | | | | |
| Very dissatisfied  1 | Dissatisfied  2 | Neither satisfied nor dissatisfied  3 | Satisfied  4 | Very  Satisfied  5 |
| 18. (F12.4) Are you satisfied with your working ability? | | | | |
| Very dissatisfied  1 | Dissatisfied  2 | Neither satisfied nor dissatisfied  3 | Satisfied  4 | Very  Satisfied  5 |
| 19. (F6.3) Are you satisfied with yourself? | | | | |
| Very dissatisfied  1 | Dissatisfied  2 | Neither satisfied nor dissatisfied  3 | Satisfied  4 | Very  Satisfied  5 |
| 20. (F13.3) Are you satisfied with your interpersonal relationship? | | | | |
| Very dissatisfied  1 | Dissatisfied  2 | Neither satisfied nor dissatisfied  3 | Satisfied  4 | Very  Satisfied  5 |
| 21. (F15.3) Are you satisfied with your sex life? | | | | |
| Very dissatisfied  1 | Dissatisfied  2 | Neither satisfied nor dissatisfied  3 | Satisfied  4 | Very  Satisfied  5 |
| 22. (F14.4) Are you satisfied with the support you get from your friends? | | | | |
| Very dissatisfied  1 | Dissatisfied  2 | Neither satisfied nor dissatisfied  3 | Satisfied  4 | Very  Satisfied  5 |
| 23. (F17.3) Are you satisfied with the conditions of your residence? | | | | |
| Very dissatisfied  1 | Dissatisfied  2 | Neither satisfied nor dissatisfied  3 | Satisfied  4 | Very  Satisfied  5 |
| 24. (F19.3) Are you satisfied with the convenience of health care services? | | | | |
| Very dissatisfied  1 | Dissatisfied  2 | Neither satisfied nor dissatisfied  3 | Satisfied  4 | Very  Satisfied  5 |
| 25. (F23.3) Are you satisfied with your traffic? | | | | |
| Very dissatisfied  1 | Dissatisfied  2 | Neither satisfied nor dissatisfied  3 | Satisfied  4 | Very  Satisfied  5 |
| The following questions are about how often you have experienced something in the past two weeks. | | | | |
| 26. (F8.1) Do you have any negative feelings? (e.g. depression, despair, anxiety, melancholy) | | | | |
| Not at all  1 | Seldom  2 | Sometimes  3 | Always  4 | Quite frequently  5 |
| Three more questions: | | | | |
| 101. Does family friction affect your life? | | | | |
| Not at all  1 | Seldom  2 | Sometimes  3 | Always  4 | Quite frequently  5 |
| 102. How about your appetite? | | | | |
| Very bad  1 | Bad  2 | Not good  3 | Good  4 | Very good  5 |
| 103. If you are asked to give a total score for your quality of life based on the above aspects (physical health, mental health, social relations and surrounding environment), how much will you give? (full score is 100 points) points | | | | |

Did you complete this questionnaire with the help of others? □ yes □ no,

How long did it take you to complete this questionnaire? （ ） minutes

1. **Diabetic Quality of Life (DQOL)**

(In reference to *Methods and Application of Quality of Life Measurement*, edited by Jiqian Fang, Beijing Medical University Press, 2000)

Filling Instructions

The following questions are to know how you feel about your quality of life, health status and daily activities. Please be sure to answer all the questions. If you are not sure how to answer a question, choose the answer closest to your true feelings.

Please answer all questions according to your own standards, wishes or feelings. Note that all problems are only your situation in the last two weeks.

Question: “In general, how much damage does diabetes mellitus bring to your health?”

Below it are five choices ranked by degree.

| Not at all  1 | Slightly  2 | Moderately  3 | Fairly  4 | Severely  5 |
| --- | --- | --- | --- | --- |

**DQOL** Table B1

| If you think diabetes mellitus is severely damaging your health, put a “〓” at the number under the “Severely”; if you think it is slightly damaging your health, put a “〓” at the number under the “Slightly”. Please note that all questions are only about how you feel in the last two weeks. | | | | |
| --- | --- | --- | --- | --- |
| 1. The effect of diabetes mellitus on physiological function: | | | | |
| D1. G1 In general, does diabetes mellitus have any harm to your health? | | | | |
| Not at all  1 | Slightly  2 | Moderately  3 | Fairly  4 | Severely  5 |
| D1. F1. 1. Do you feel anything uncomfortable such as skin itching, limb numbness and pain? | | | | |
| Not at all  1 | Seldom  2 | Sometimes (half of the time)  3 | Always  4 | Quite frequently  5 |
| D1. F1. 2. Does the feeling of physical discomfort interfere with your personal life? | | | | |
| Not at all  1 | Slightly  2 | Moderately  3 | Fairly  4 | Severely  5 |
| D1. F1.3. Do you feel it is harder and harder to looking at things? | | | | |
| Not at all  1 | Seldom  2 | Sometimes (half of the time)  3 | Always  4 | Quite frequently  5 |
| D1. F1. 4. Do you find it more and more difficult to listen to others? | | | | |
| Not at all  1 | Seldom  2 | Sometimes (half of the time)  3 | Always  4 | Quite frequently  5 |
| D1. F1. 5. Does your hearing have a negative impact on your daily life? | | | | |
| Not at all  1 | Slightly  2 | Moderately  3 | Fairly  4 | Severely  5 |
| D1. F1. 6. Does your vision have a negative impact on your daily life? | | | | |
| Not at all  1 | Slightly  2 | Moderately  3 | Fairly  4 | Severely  5 |
| D1. F1. 7. Do you feel chest pain, tightness and palpitations? | | | | |
| Not at all  1 | Seldom  2 | Sometimes (half of the time)  3 | Always  4 | Quite frequently  5 |
| D1. F1. 8. Is your life adversely affected by skin or foot diseases? | | | | |
| Not at all  1 | Slightly  2 | Moderately  3 | Fairly  4 | Severely  5 |
| D1. F1. 9. Has your ability to respond to external things decreased? | | | | |
| Not at all  1 | Slightly  2 | Moderately  3 | Fairly  4 | Severely  5 |
| D1. F1. 10. Do you feel hungry? | | | | |
| Not at all  1 | Seldom  2 | Sometimes (half of the time)  3 | Always  4 | Quite frequently  5 |
| D1. F1. 11. Do you feel thirsty and frequently want to urinate? | | | | |
| Not at all  1 | Seldom  2 | Sometimes (half of the time)  3 | Always  4 | Quite frequently  5 |
| 2. The impact of diabetes mellitus on psychological/mental health: | | | | |
| D1. G2. Does diabetes mellitus bring trouble and inconvenience to your daily life? | | | | |
| Not at all  1 | Seldom  2 | Sometimes (half of the time)  3 | Always  4 | Quite frequently  5 |
| D1. F2. 1. What do you think diabetes mellitus means to you? | | | | |
| Not at all  1 | Seldom  2 | Sometimes (half of the time)  3 | Always  4 | Quite frequently  5 |
| D2. F2. 1. Are you worried that you will die suddenly? | | | | |
| Not at all  1 | Seldom  2 | Sometimes (half of the time)  3 | Always  4 | Quite frequently  5 |
| D2. F3. 1. Does dietary control bother you? | | | | |
| Not at all  1 | Seldom  2 | Sometimes (half of the time)  3 | Always  4 | Quite frequently  5 |
| D2. F3. 2. Is it troublesome for you to test your urine sugar regularly or check your blood sugar in the hospital? | | | | |
| Not at all  1 | Seldom  2 | Sometimes (half of the time)  3 | Always  4 | Quite frequently  5 |
| D2. F4. Do you feel nervous or embarrassed? | | | | |
| Not at all  1 | Seldom  2 | Sometimes (half of the time)  3 | Always  4 | Quite frequently  5 |
| D2. F5. 1. Are you satisfied with your current treatment effect? | | | | |
| Very  Satisfied  1 | Satisfied  2 | Neither satisfied nor dissatisfied  3 | Dissatisfied  4 | Very dissatisfied  1 |
| D2. F5. 2. Do you believe you can overcome the trouble of disease? | | | | |
| Not at all  1 | Seldom  2 | Moderately  3 | Fairly  4 | Absolutely  5 |
| 3. The impact of diabetes mellitus on social relations: | | | | |
| D3. G3. In general, does diabetes mellitus affect your interpersonal relationship? | | | | |
| Not at all  1 | Slightly  2 | Moderately  3 | Fairly  4 | Severely  5 |
| D3. F6. 1. Are you being rejected because of diabetes mellitus? | | | | |
| Not at all  1 | Seldom  2 | Sometimes (half of the time)  3 | Always  4 | Quite frequently  5 |
| D3. F6. 2. Does diabetes mellitus affect your status and role in your family or in your unit? | | | | |
| Not at all  1 | Slightly  2 | Moderately  3 | Fairly  4 | Severely  5 |
| D3. F7. Have you communicated with other patients about diabetes mellitus experience, knowledge and problems? | | | | |
| Not at all  1 | Seldom  2 | Sometimes (half of the time)  3 | Always  4 | Quite frequently  5 |
| 4. Impact of treatment on patients: | | | | |
| D4. F8. Do you have any adverse drug reactions such as allergy and nausea? | | | | |
| Not at all  1 | Seldom  2 | Sometimes (half of the time)  3 | Always  4 | Quite frequently  5 |
| D4. F8. Do you have hypoglycemic reactions such as palpitation, dizziness and sweating? | | | | |
| Not at all  1 | Seldom  2 | Sometimes (half of the time)  3 | Always  4 | Quite frequently  5 |
| D4. F3. 3. Does dietary control have restrictions on your lifestyle or habits? | | | | |
| Not at all  1 | Slightly  2 | Moderately  3 | Fairly  4 | Severely  5 |

Did you complete this questionnaire with the help of others? □ yes □ no,

How long did it take you to complete this questionnaire? （ ） minutes
